# Enhancing Homeopathic Prescribing for Chronic Cough by Classifying the Reliability of Polar Symptoms

**DOI:** 10.1055/a-2631-9180

**Published:** 2025-10-23

**Authors:** Harleen Kaur, Lex Rutten, José E. Eizayaga, Shalini Rao, Anurag Bajpai, Chetna Deep Lamba, Jyoti Sachdeva, Vinitha ER, Sonia Raizada, Rompicherla G.R. Kiranmayee, Bodankar Rajashekhar, Chittaranjan Kundu, Vaishali Shinde, Sujata Choudhury, Amulya Ratna Sahoo, Ratan Chandra Shil, Abhijit Chakma, Nidhi Mahajan, Alok Mishra, Anil Khurana, Praveen Oberai, Raj K. Manchanda

**Affiliations:** 1 Central Council for Research in Homoeopathy, New Delhi, India; 2 Independent Researcher, Breda, The Netherlands; 3 Department of Homeopathy, Maimonides University, Buenos Aires, Argentina; 4 National Homoeopathy Research Institute in Mental Health (NHRIMH), Kottayam, Kerala, India; 5 Dr. D.P. Rastogi Central Research Institute (Homoeopathy), Noida, Uttar Pradesh, India; 6 Extension Clinical Research Unit of DSU, Hyderabad, Andhra Pradesh, India; 7 Regional Research Institute for Homoeopathy, Gudivada, Krishna Dist., Andhra Pradesh, India; 8 Dr. Anjali Chatterji Regional Research Institute of Homoeopathy, Kolkata, West Bengal, India; 9 Regional Research Institute (Homoeopathy), Navi Mumbai, Maharashtra, India; 10 Regional Research Institute (H), Puri, Odisha, India; 11 Drug Proving Unit, Dr. Abhin Chandra Homoeopathic Medical College and Hospital, Unit-III, Bhubaneswar, Odisha, India; 12 Regional Research Institute for Homoeopathy, Agartala, Tripura, India; 13 Central Research Institute for Homoeopathy, MPK Homoeo Medical College, Jaipur, Rajasthan, India

**Keywords:** prognostic factor research, cumulative binomial probability, chronic cough, statistical variation

## Abstract

**Background:**

Assessment of relationship between polar symptoms (PS), such as weather, activity or food, and homeopathic medicines in chronic cough has produced some confusing outcomes. Statistical variation in the data partly explains the seemingly conflicting outcomes in prognostic factor research (PFR) of PS. Classification of statistical probability and selection of statistically reliable data can reduce conflicting outcomes.

**Objective:**

To make an inventory of statistically reliable PS and their relationship with homeopathic medicines in chronic cough.

**Methods:**

A prospective observational cohort study was conducted at 10 outpatient centers of the Central Council for Research in Homoeopathy, India. Patients with chronic cough were followed for 12 months. Effect of treatment was assessed using the ORIDL (Outcome in Relation to Impact on Daily Living) instrument, with symptom severity and health status evaluated respectively via the Cough Severity Index and EuroQoL 5D-5L. Prevalence and likelihood ratios (LRs) of PS were calculated for general and medicine-specific populations. Cumulative binomial probability (CBP) was used to determine whether symptom prevalence among good responders to specific medicines significantly differed from that of the general population.

**Results:**

Some LRs were unexpectedly low, which could be explained by asymmetry of frequency distributions, resulting in high prevalence of one symptom pole. CBP for homeopathic medicines was subsequently performed, with 131 symptoms classified as ‘certain’ or ‘probable’: 21 symptoms for *Arsenicum album* , 23 for *Bryonia* , 11 for *Calcarea carbonica* , 3 for *Lycopodium* , 15 for *Natrium muriaticum* , 5 for *Nux vomica* , 17 for *Phosphorus* , 13 for *Pulsatilla* , 10 for *Silicea* , and 13 for *Sulphur* .

**Conclusion:**

Conflicting outcomes in PFR of PS can partly be explained by statistical variation. A classification of reliability was not feasible when expressed using the classical 95% confidence interval but could be achieved by CBP, resulting in certain and probable PS for 10 medicines. We still need clinical expertise and corroboration for future research. Practitioners need scientific training to prevent bias and use research instruments such as Likert scales and questionnaires.

## Introduction

Cough is one of the most common symptoms for which people present to primary care and is a chief complaint for patients seeking medical attention in respiratory or allergy specialist clinics. In clinical guidelines, the definition of chronic cough is one persisting in a patient for more than 8 weeks; however, in many epidemiological studies, chronic cough is defined as one that lasts longer than 3 months. The definition of chronic cough is guided by expert opinion, as definitive clinical criteria to distinguish acute from chronic cough are lacking. 1 Chronic cough poses a major public health challenge: a pooled analysis of 90 different studies found the overall prevalence to be 9.6%. 2

Chronic cough is a symptom associated with various conditions, including asthma, rhinitis, post-nasal drip, and gastroesophageal reflux disease (GERD). The medical community faces considerable challenges in diagnosing and treating patients with the symptom. These difficulties stem primarily from an incomplete understanding of the underlying causes and pathological mechanisms involved in chronic cough as well as a lack of effective treatment options. These limitations in our current knowledge and therapeutic approaches make chronic cough a complex issue for both patients and health care providers, highlighting the need for further research and development in this area.

Homeopathy is often used to treat chronic cough. The choice of the most suited homeopathic medicine is, among others, determined by the individual's reaction to all kinds of influences (modalities) such as weather, activity and food. Many modalities can have either positive or negative effects on the complaint and/or the patient's general well-being and are therefore called ‘polar symptoms’ (PS). The representation of PS is often problematic because the same medicine is mentioned in both opposites of the same modality because of statistical variation. For instance: most *Arsenicum* patients are chilly, but some are not. The proper way to assess the relationship of both poles of PS and the effect of specific homeopathic medicines is prognostic factor research (PFR).

Prospective PFR has been performed by the Central Council for Research in Homoeopathy (CCRH) in India. 3 4 5 Kaur et al focused on identifying the effectiveness of homeopathic medicines for treating chronic cough and emphasized the importance of PS in treatment. 4 It compared the outcomes of three medicines ( *Phosphorus* , *Pulsatilla* , *Sulphur* ) in clinical cases and assessed how PS can improve treatment approaches. The study reflected on discrepancies in data compared with existing studies and suggested prioritising PS for better outcomes. Scores were recorded using the Leicester Cough Questionnaire, along with EuroQoL (EQ)-5D-5L, and additional assessment using a Physician Assessment Scale.

Traditional repertories in homeopathy often rely on the absolute frequency of symptom occurrences in populations responding well to specific medicines. This approach tends to favor medicines addressing common symptoms. However, a more refined method would utilize relative frequency or symptom prevalence instead. This shift aligns with Bayesian principles, allowing us to update our practices based on emerging evidence.

The strength of a relationship between a specific symptom and a particular medicine can be expressed as likelihood ratio (LR).

LR = (prevalence of the symptom in the medicine population) / (prevalence of the symptom in the remainder of the population).

If LR > 1 the symptom is an indication for the medicine: i.e., the chance increases that the medicine works. If LR < 1, the symptom is a contraindication.

The LR is a quantitative outcome of the relationship between symptom and medicine, but it does not express the statistical certainty of the outcome. If the population is small, the statistical certainty is lower than in a large population, but the LR can be the same in both cases.

Hence, the PFR on chronic cough has produced some contradictory results. Statistical analysis indicated statistical variation as the most probable cause. When calculating LRs of all symptoms for all medicines, we find symptoms that are related to specific medicines, but also symptoms that are not. 4 The LR values of unrelated symptoms are mostly not exactly 1.0 but vary around unity to a degree that depends mostly on the sample size of each medicine population.

Usually, statistical uncertainty is expressed by the 95% confidence interval (CI). However, the outcome can be less certain yet still useful in daily medical practice; a 90% CI or 80% CI, for example, could also be useful. Therefore, the need was felt for a statistical technique to assess the reliability of a symptom for prescription, even if the CI is found to be low, say 80%. The cumulative binomial probability (CBP) offers a practical approach by calculating the binomial probability that the prevalence of the symptom in the medicine population is the same as in the remainder of the population. 6 If CBP is close to 1.0, there is high statistical certainty that the prevalence of the symptom in the medicine population is higher than in the remainder of the population: this is equivalent to the statistical certainty that LR > 1.

CBP offers a useful classification of statistical certainty. For PS, we also expect that the symptom is corroborated by its opposite: this is classified by a difference in CBP of opposite poles. This classification enables the categorization of symptoms as ‘Certain’ or ‘Probable,’ thereby enhancing the precision of homeopathic prescriptions. In the present study, our primary objective was to make an inventory of statistically reliable PS and their relationship with specific homeopathic medicines in the treatment of chronic cough.

Previously, ‘Centre of Mass’ and ‘Leverage’ were proposed as measures comparable to polarity analysis, including comparison with the remainder of the population. 3 However, statistical variation appeared to be the cause of conflicting outcomes. These measures do not express statistical variation and were thus abandoned for the current investigation.

## Methods

A prospective observational cohort study was conducted on the chronic cough patients attending the outpatient departments of 10 centers of the CCRH, India: Dr. D. P. Rastogi Central Research Institute (H), Noida; National Homoeopathy Research Institute in Mental Health, Kottayam; Drug Standardization Unit, Hyderabad; Regional Research Institute for Homoeopathy, Mumbai; Dr. A. C. Regional Research Institute for Homoeopathy, Kolkata; Clinical Research Unit (H), Agartala; Regional Research Institute for Homoeopathy Extn., Puri; Drug Proving Unit, Bhubaneswar; Central Research Institute for Homoeopathy, Jaipur; and Regional Research Institute for Homoeopathy, Gudivada. The study lasted from May 2018 to December 2020.

Homeopathic physicians, postgraduates in homeopathy, with 5 to 10 years of professional experience, and employed as Research Officers in the participating research centers, were involved as investigators. All of them were trained in the study protocol, case taking, the filling in of a Likert scale-based questionnaire, and were oriented as per the expected outcomes before the initiation of the study.

Participants aged 7 to 65 years with a cough lasting over 8 weeks were included in the study. Exclusions applied to those who had taken antibiotics in the past week, had hemoptysis, signs of foreign body aspiration, uncontrolled hypertension, or were on medications such as ACE inhibitors known to cause cough. Participants were allowed to continue other conventional treatments. Smokers, substance abusers, those with interfering health conditions, and pregnant or lactating women were also excluded from the study. All procedures followed proper ethical standards and with the Declaration of Helsinki of 1975 as revised in 2013. Necessary clearance by the 20 ^th^ meeting of the Institutional Ethical Committee of the CCRH, Ref No.1-3/201617/CCRH/Tech./20th EC/3242, dated February 14, 2017, was obtained. The trial was registered with the Clinical Trial Registry of India (CTRI/2018/05/013973) on May 18, 2018.

Initially, participants were screened based on age and cough duration, followed by assessment for three specific conditions: upper airway cough syndrome, GERD-related cough, and asthma-related syndromes. Written informed consent was obtained from patients or guardians (for minors), with additional assent for those aged 7 to 17 years. A detailed case history was recorded using a structured proforma. For patients who were young or who had limited responding abilities, clinical data were assessed verbally with the help of accompanying attendants. Based on the case history and the set of symptoms elicited during the personal consultation provided to each patient, an individualized homeopathic medicine was prescribed to the participant.

Patients received usual treatment, but during the first consultation a questionnaire with 30 polar general and 27 polar cough symptoms, structured as 7-point Likert scales, 7 was completed with the help of a practitioner. Each 7-point scale covered a range of intensities for the influence of a given modality: for instance, ‘Aggravation stronger than in most people’—‘Strong aggravation’—‘Aggravation’—‘No influence’—‘Amelioration’—‘Strong amelioration’—‘Amelioration stronger than in most people’.

There were no restrictions regarding the prescription of medicines. Anonymized patient data, presence of symptoms and treatment outcome data were first recorded in a paper record sheet and subsequently entered in a pre-formatted spreadsheet. The patients were followed up every 2 weeks during the first 8 weeks, then every 4 weeks or up to 12 months after inclusion. In cases where the patient stopped the ongoing homeopathic treatment before the fourth follow-up, the last set of data for the patient was carried forward, as per the modified intention-to-treat method. The effect of treatment was evaluated at each follow-up with the ORIDL (Outcome in Relation to Impact on Daily Living) instrument. 8

The patients visiting the outpatient departments were initially screened by the investigator present on site. Patients matching the exclusion criteria were excluded and, if necessary, referred. If the diagnosis of a chronic cough was not established, patients received usual care and completed the following assessments: ORIDL, Cough Severity Index (CSI), EQ-5D-5L, and a cough-specific and a homeopathic questionnaire for general symptoms.

If a diagnosis of chronic cough was confirmed and the patient gave informed consent, he or she was enrolled in the follow-up study. Each follow-up visit included repeated assessments using the above tools. If, during the process, a patient's ORIDL score was ≥2 (indicating a meaningful improvement), they continued under regular protocol-based follow-up. The last follow-up visit also included final assessments using ORIDL, CSI and EQ-5D-5L to evaluate outcomes and track patient progress over the follow-up period.

Whilst the ORIDL outcomes were used as a reference for selecting cases for the calculation of PFR in this work, the other two tools were used to assess the improvement in the cough-specific parameters, the outcomes of which are beyond the scope of this paper and have already been reported elsewhere. 5

Cases with an ORIDL score of at least +2 at the last follow-up were selected for calculations of LR and CBP if they had been prescribed any one of the 10 most frequently used medicines: i.e., *Arsenicum album (Ars), Bryonia (Bry), Calcarea carb (Calc), Lycopodium (Lyc), Natrium mur (Nat-m), Nux vomica (Nux-v), Phosphorus (Phos), Pulsatilla (Puls), Silicea (Sil)* or *Sulphur (Sulph)* . The medicines that had LR > 1 were then selected for further analysis of reliability through CBP.

**Fig. 1:**
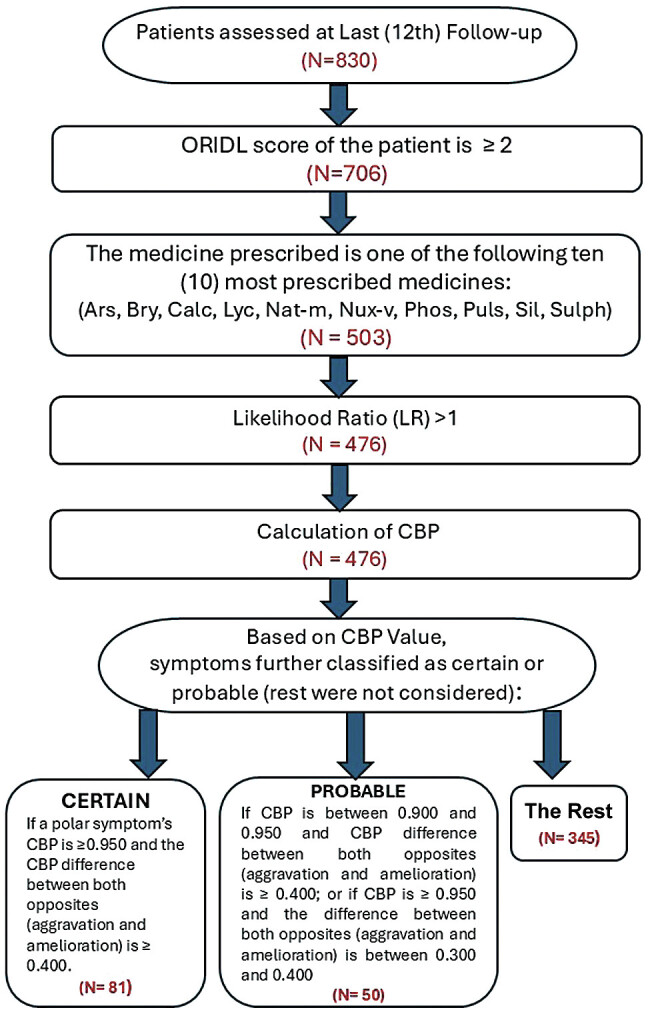
Flowchart illustrating the procedure for determining CBP-based Certain and Probable symptoms. CBP, cumulative binomial probability.

The PS with LR > 1 were picked for sufficient degree of statistical certainty using CBP. The calculation of CBP was performed using the ‘binomial distribution’ function of Microsoft Excel. LRs were calculated with a custom-made formula. A flowchart explaining this procedure is presented in Fig. 1 .

CBP was therefore applied to calculate the statistical probability that the prevalence of the symptom in the respective medicine population (the population responding well to a specific medicine) was the same as in the remainder of the population. 6 For each given medicine, our preliminary choices for the classification of the outcome with CBP were:

1. Certain outcome: If a polar symptom's CBP is ≥0.950 and the CBP difference between both opposites (aggravation and amelioration) is >0.400.
2. Probable outcome: If CBP is between 0.900 and 0.950 and the CBP difference between both opposites (aggravation and amelioration) is >0.400; or if CBP is ≥0.950 and the difference between both opposites (aggravation and amelioration) is between 0.300 and 0.400.

## Results

The final database consisted of 830 cases collected from various institutes under CCRH. The contributions from each institute were as follows: National Homoeopathy Research Institute in Mental Health, Kottayam—153 cases; Dr. D.P. Rastogi Central Research Institute (Homoeopathy), Noida—127 cases; Extension Clinical Research Unit of DSU, Hyderabad—112 cases; Dr. Anjali Chatterji Regional Research Institute of Homoeopathy, Kolkata—88 cases; Regional Research Institute for Homoeopathy, Gudivada—85 cases; Regional Research Institute for Homoeopathy, Agartala—69 cases; Regional Research Institute (Homoeopathy), Navi Mumbai—64 cases; Regional Research Institute (Homoeopathy), Puri—63 cases; Drug Proving Unit, Bhubaneswar—57 cases; and Central Research Institute for Homoeopathy, Jaipur—12 cases.

Out of the 830 cases gathered by the respective institutes, only those with an ORIDL score of +2 or higher were selected, reducing the total number of cases to 706. From these, only the cases involving prescriptions of the 10 most prescribed medicines were included in the final analysis, resulting in a total of 503 cases ( Fig. 1 ). The distribution of these cases across the medicines was as follows: *Ars* —105, Bry—17, *Calc* —23, *Lyc* —31, *Nat-m* —22, *Nux-v* —21, *Phos* —122, *Puls* —77, *Sil* —23, and *Sulph* —62. When considering cases with LR ≥ 1, the number further reduced to 476. Based on the CBP classification, 81 symptoms were identified as ‘Certain’, 50 as ‘Probable’, and the remaining 345 fell into 'The rest' category.

**Fig. 2:**
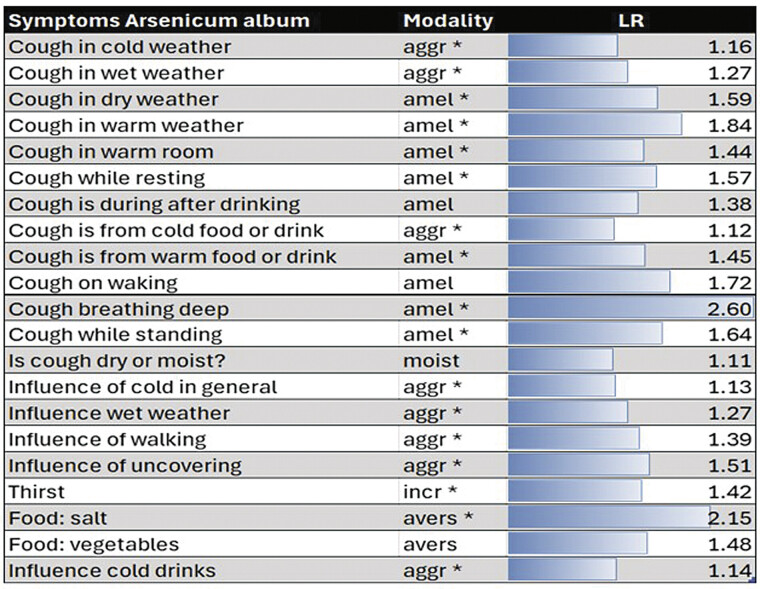
Outcome of PFR along with modalities for *Arsenic album* ; only symptoms that are classified as ‘Certain’ or ‘Probable’ are shown. The strength of LR is indicated by data bars and * indicates that this LR outcome is statistically ‘Certain’. LR, likelihood ratio; PFR, prognostic factor research.

Results for each of the 10 homeopathic medicines are shown in Figs. 2 3 4 5 6 7 8 9 10 11 . These figures show the symptom, the modality (amelioration/desire or aggravation/aversion) and the LR. An asterisk after the modality indicates that the symptom was classified as ‘Certain’; the other symptoms were classified as ‘Probable’. The length of the color bars in the LR column indicates the relative importance of the symptom for the medicine. For instance, the strongest indication for *Ars* is the symptom ‘Cough ameliorated by breathing deep’, with LR = 2.60 ( Fig. 2 ).

**Fig. 3:**
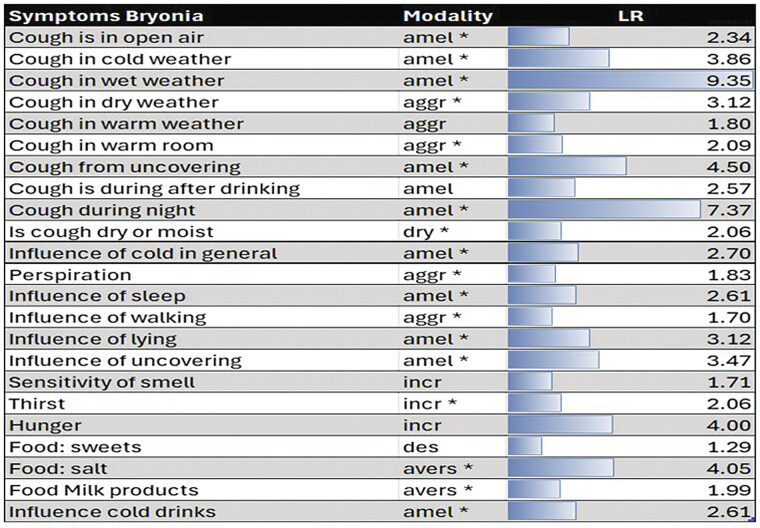
Outcome of PFR along with modalities for *Bryonia* ; only symptoms that are classified as ‘Certain’ or ‘Probable’ are shown. The strength of LR is indicated by data bars and * indicates that this LR outcome is statistically ‘Certain’. LR, likelihood ratio; PFR, prognostic factor research.

**Fig. 4:**
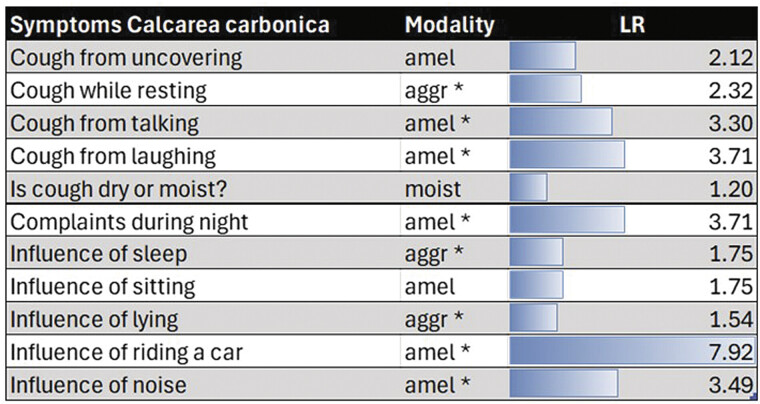
Outcome of PFR along with modalities for *Calcarea Carb* ; only symptoms that are classified as ‘Certain’ or ‘Probable’ are shown. The strength of LR is indicated by data bars and * indicates that this LR outcome is statistically ‘Certain’. LR, likelihood ratio; PFR, prognostic factor research.

**Fig. 5:**
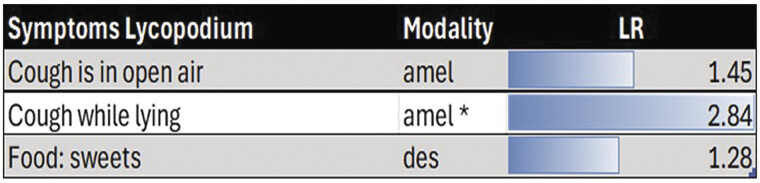
Outcome of PFR along with modalities for *Lycopodium* ; only symptoms that are classified as ‘Certain’ or ‘Probable’ are shown. The strength of LR is indicated by data bars and * indicates that this LR outcome is statistically ‘Certain’. LR, likelihood ratio; PFR, prognostic factor research.

**Fig. 6:**
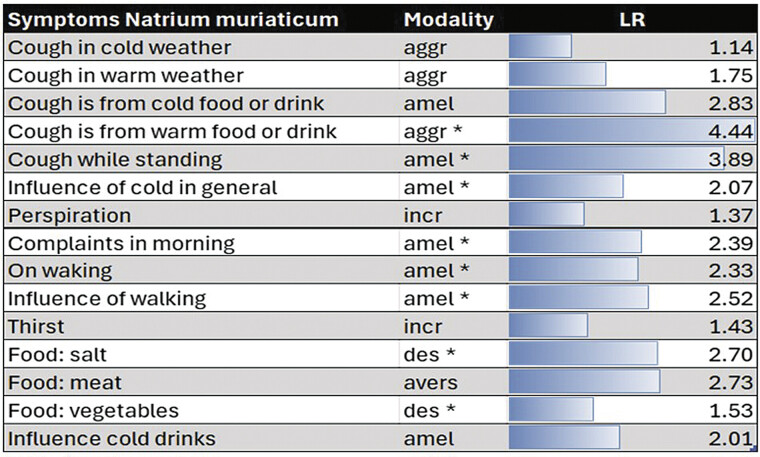
Outcome of PFR along with modalities for *Natrium muriaticum* ; only symptoms that are classified as ‘Certain’ or ‘Probable’ are shown. The strength of LR is indicated by data bars and * indicates that this LR outcome is statistically ‘Certain’. LR, likelihood ratio; PFR, prognostic factor research.

**Fig. 7:**
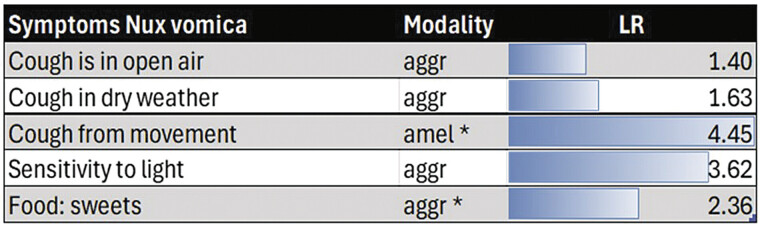
Outcome of PFR along with modalities for *Nux vomica* ; only symptoms that are classified as ‘Certain’ or ‘Probable’ are shown. The strength of LR is indicated by data bars and * indicates that this LR outcome is statistically ‘Certain’. LR, likelihood ratio; PFR, prognostic factor research.

**Fig. 8:**
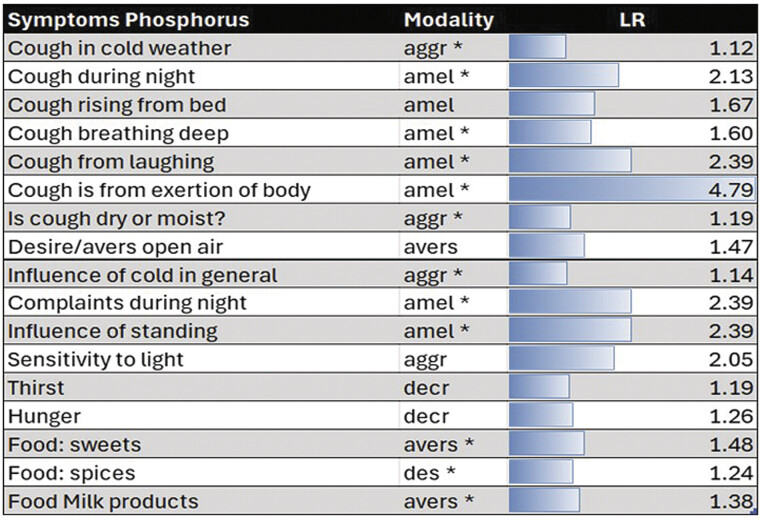
Outcome of PFR along with modalities for *Phosphorus* ; only symptoms that are classified as ‘Certain’ or ‘Probable’ are shown. The strength of LR is indicated by data bars and * indicates that this LR outcome is statistically ‘Certain’. LR, likelihood ratio; PFR, prognostic factor research.

**Fig. 9:**
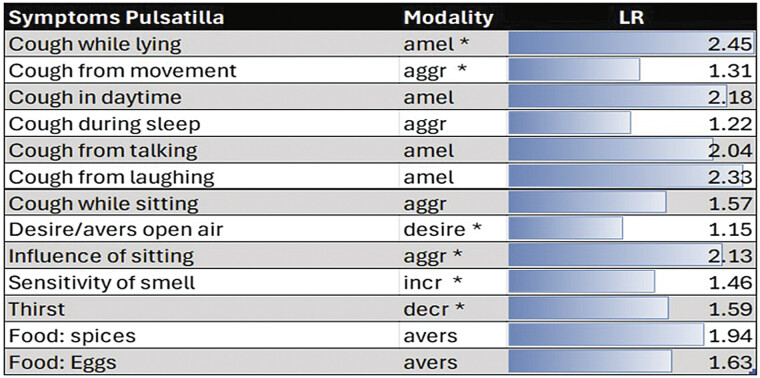
Outcome of PFR along with modalities for *Pulsatilla* ; only symptoms that are classified as ‘Certain’ or ‘Probable’ are shown. The strength of LR is indicated by data bars and * indicates that this LR outcome is statistically ‘Certain’. LR, likelihood ratio; PFR, prognostic factor research.

**Fig. 10:**
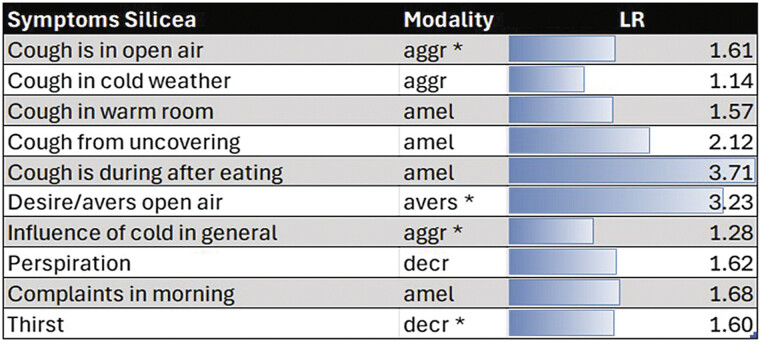
Outcome of PFR along with modalities for *Silicea* ; only symptoms that are classified as ‘Certain’ or ‘Probable’ are shown. The strength of LR is indicated by data bars and * indicates that this LR outcome is statistically ‘Certain’. LR, likelihood ratio; PFR, prognostic factor research.

**Fig. 11:**
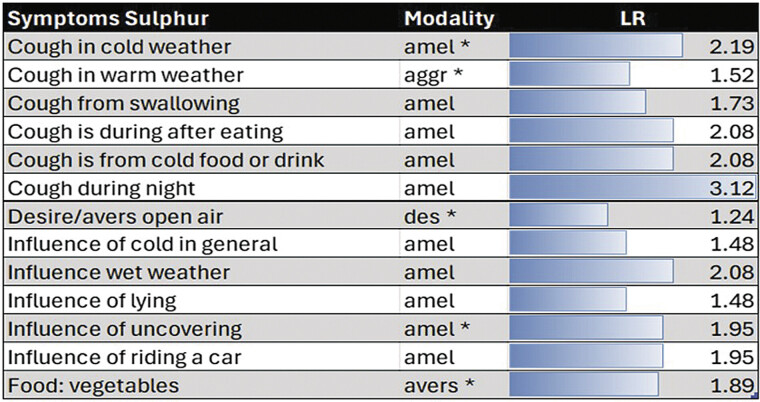
Outcome of PFR along with modalities for *Sulphur* ; only symptoms that are classified as ‘Certain’ or ‘Probable’ are shown. The strength of LR is indicated by data bars and * indicates that this LR outcome is statistically ‘Certain’. LR, likelihood ratio; PFR, prognostic factor research.

To further illustrate the classification, *Bry* was classified as ‘Certain’ for alleviating cough in cold weather (LR = 3.86) ( Fig. 3 ), whilst *Ars* showed certainty for aggravation in similar conditions (LR = 1.16; Fig. 2 ). Commonly observed symptoms included ‘Cough in cold weather’ and ‘Influence of cold in general’, which appeared in 6 of the 10 medicines ( Figs. 2 , 3 , 6 , 8 , 10 , 11 ). Some symptoms are ‘Certain’ despite low LR, and others are ‘Probable’ despite relatively high LR—this matter is explained in Discussion.

Summarizing the outcome, *Ars* had 21 ‘Certain’ or ‘Probable’ symptoms, 17 of them classified as ‘Certain’ ( Fig. 2 ); *Bry* 23 symptoms, 18 of them ‘Certain’ ( Fig. 3 ); *Calc* 11 symptoms, 8 of them ‘Certain’ ( Fig. 4 ); *Lyc* 3 symptoms, 1 of them ‘Certain’ ( Fig. 5 ); *Nat-m* 15 symptoms, 8 of them ‘Certain’ ( Fig. 6 ); *Nux-v* 5 symptoms, 2 of them ‘Certain’ ( Fig. 7 ); *Phos* 17 symptoms, 12 of them ‘Certain’ ( Fig. 8 ); *Puls* 13 symptoms, 6 of them ‘Certain’ ( Fig. 9 ); *Sil* 10 symptoms, 4 of them ‘Certain’ ( Fig. 10 ); and *Sulph* 13 symptoms, of which 5 were found to be ‘Certain’ ( Fig. 11 ).

## Discussion

Our findings combine each LR with its CBP of a symptom prevalence in the medicine population as compared with the rest of the population: i.e., when LR ≥ 1. Specifically for PS, we can combine the CBP difference between opposite poles to show corroboration by the opposite pole. This interpretation supersedes the earlier threshold of LR ≥ 1.5 as a sole sign of statistical certainty.

Using this method, we came across several instances where LRs of 1.0 to 1.5 were statistically significant and LRs ≥ 1.5 were not. For example, ‘Aggravation of cough from cold food or drink’ as an indication for *Ars* (LR = 1.12) is ‘Certain’ in statistical terms. This is caused not only by a larger sample size of the *Ars* population ( *n* = 105), but also by the asymmetry of the PS: many more patients had aggravation than amelioration, further increasing the sample size for this pole. Conversely, the considerably greater LR = 2.57 for ‘Improvement of cough after drinking’ as an indication for *Bry* is ‘Probable’ due to the smaller population of *Bry* ( *n* = 17) in our study. Here the certainty for one pole is also not supported by sufficient CBP difference with the other pole (<0.40), which reduces the statistical classification to ‘Probable’.

This finding demonstrates that assessment of variation is an essential part of PFR: LR values are incomplete without their statistical certainty. It should be made clear that CBP expresses only statistical noise and does not preclude the presence of bias. For example, the low LRs of cold-related symptoms for *Ars* will seem strange to practitioners, since cold aggravation is a well-known fact in the Materia Medica and practitioners' experience as a sign for *Ars* . However, a closer examination of the data reveals likely bias: the frequency distributions are extremely asymmetrical, the highest frequency being for ‘strong aggravation’ rather than neutral. The high prevalence of one pole causes a high prevalence of that pole in the remainder of the population and thus low LRs, caused by the ‘ceiling effect’. LR cannot be higher than one divided by the prevalence in the remainder of the population: for instance, if the prevalence in the remainder of the population is 50%, the maximum LR = 2.0.

A duplication of such studies involving clinical judgment is thus one of the research priorities. The converse of low LRs due to bias is high LRs due to (confirmation) bias. Confirmation bias can be identified when there are high LRs for a symptom in one or a few medicines, but there are low LRs for that particular symptom in other medicines. This situation occurs because the symptom is more readily observed if it fits the medicine; it often happens for example in keynote symptoms for particular medicines.

No clear signs of confirmation bias can be seen from this database. The only high LRs, ‘Cough > in wet weather’ ( *Bry* , LR = 9.35) and ‘Riding in a car >’ ( *Calc* , LR = 7.92), could be statistical outliers among this large dataset. A strong point favoring the results is the comparability of our data with various independent databases. Our current database pertains to chronic cases, the other database to acute (coronavirus disease 2019 [COVID-19]) cases. 9 Both these databases are prospective in design.

One interesting instance of outcome contrary to information in Kent's repertory is the polar symptom 'Thirst increased/decreased' for *Phos* . The repertory has some rubrics showing that the *Phos* patient feels thirsty: 'Thirst' (bold type), 'Thirst for large quantities' (bold type) and 'Desire cold drinks' (bold type). There is also *Phos* in the rubric 'Thirstless', but in ordinary type. In the COVID-19 database, LR = 1.19 for *Phos* and 'Increased thirst'; CBP = 0.898 (Possible). In the chronic cough database, LR = 1.19 for *Phos* and 'Decreased thirst'; CBP = 0.940 (Probable). The PFR outcome for the symptom 'Thirst' as evaluated in 208 *Phos* cases renders the current entry in the rubric 'Thirst' improbable. As mentioned, the potential for improvement of homeopathy data is vast. Yet we can be hopeful for improving this huge dataset through the lens of PFR, especially the PS, which often forms the strong basis of a prescription. PFR especially improves the reliability of the most common symptoms and therefore greatly enhances the reliability of homeopathic prescriptions.

The questionnaire with 7-point Likert scales was designed to improve the assessment of symptoms. 7 However, after that study was completed, it was realised that patients sometimes face difficulty in grading a symptom on a Likert scale. That realisation necessitated more involvement of the investigator in eliciting the symptoms or its interpretation on the Likert scale. This approach may have led to some bias in marking the grades for the symptom.

We can anticipate the gradual development of homeopathy-related PFR. Access to data for comparisons will necessitate an autonomous organization that assures the quality and accessibility of research outcome. The quality of data will be improved largely through scientific training of all participating homeopathic practitioners since the quality of cases depends mainly on the scientific capability of the practitioner.

Our work, though promising for prognostic assessment of symptoms for prescribing homeopathic medicines in chronic cough conditions, is still too early in its development to attempt to compare its outcomes and implications with PFR-related tools such as QUIPS and GRADE that have been applied to conventional medicine. 10 11 Such attempts may be made in future for a better understanding and standardization of this analytical technique.

## Conclusion

CBP enables us to recognize data with large statistical variation. This is especially useful in PS if the frequency distribution is asymmetric, with much higher prevalence of one pole. This results in much variation in the small population and opposite poles seemingly indicating the same medicine. By deleting statistically unreliable outcome of PFR, we can enhance the reliability of homeopathic prescriptions. Some LRs were lower than anticipated: this was due to asymmetrical frequency distributions with a high prevalence of one pole, causing a ‘ceiling effect’ for LR. However, corroboration by opposite and comparable symptoms confirmed the symptom as indicative for the medicine.

The homeopathic practitioner plays a vital role in the scientific process; clinical expertise is indispensable to estimate the real importance of each symptom in each individual case. On the other hand, practitioners need additional scientific training to fulfil this role. The availability of a diversity of information rectifies and necessitates corroboration from different sources.

Variation underscores the necessity for further comparative research on independent databases, especially involving statistically uncertain outcome. This should set the PFR agenda for future studies. Clinical expertise should not be ignored in PFR, especially when using Likert scales. A homeopathic symptom is relevant only when the intensity is more than average in the whole population. This can only be assessed by an experienced practitioner. All these aspects constitute the specific scientific identity of homeopathy.

## Highlights

- This work is the result of analysing data from chronic cough patients, focusing on the polar symptoms (PS) and the corresponding homeopathic indications for various medicines.
- For greater precision in clinical practice, prognostic factor research (PFR) enhances homeopathic prescriptions by integrating statistical verification.
- Likelihood ratio (LR) quantifies symptom–medicine relationships but it does not reflect statistical certainty, which decreases in smaller populations despite identical LR values.
- The classification of statistical reliability of PFR outcome is feasible by the use of cumulative binomial probability (CBP).
- CBP adds rigour to the correlation process by considering both LR and the CBP difference between opposite symptom poles, thereby improving the precision of prognostic factor assessment.
- Preventing asymmetry of PS requires clinical estimates and practitioner training, with PFR advancing through independent data comparison.
- Statistical tools alone should not guide prescriptions or treatment decisions: they must be integrated with clinical judgment for final decision-making.
